# The Nourishment of Children

**Published:** 1867-02

**Authors:** Wm. Henry Cumming, J. M. Johnson


					﻿ARTICLE II.
The Nourishment of Children.—By Wm. Henry Cumming,
M. D., with remarks by J. M. Johnson, M. D.
There is no subject that commends itself more strongly
to our attention than the one that heads this article. When
we reflect that health, constitution and life, are dependent
upon nutrition, and that most infantile diseases are, one way
and another, the result of improper food, the question is
doubly enhanced, and challenges the most earnest attention
at the hands of the medical profession at large. Nor will it
do to be informed upon this subject ourselves alone: we
must impart it to all the mothers of the land, and not only
teach it to them once, but continue to teach it to them every
day of our lives, when opportunity offers, until the laws of
development and growth are better understood and acted
upon than they are at present. Look at your statistical ta-
bles, and see the fearful inroads upon infantile life, not only
in the great cities, where bad ventillation, poor food, and
criminal dissipation and neglect cuts them off, but in the ru-
ral districts and small villages also, where, in the main,
there is an exemption from destitution and the vices that
poison the morals no less than the constitution. The readi-
ness with which the young constitution responds to reme-
dies, and urges on the process of repair, gives an earnest of
what ought to be the result where the laws of nutrition are
properly understood and carried out.
To make bone and teeth, and develop a good physical con-
stitution, capable of resisting the ordinary tendencies of dis-
ease, it seems to us is within the scope of reasonable proba-
bility, even under the disadvantage of artificial means. We
believe there is a criminal neglect that cries loundly against
us, and which, as Christians, and conservators of health and
life, we dare not longer indulge. The summer complaint in
this country, the watery gripes of England, and like diseases
all over the world, could, in our opinion, be greatly miti-
gated, and fatal results generally prevented, by a proper at-
tention to the nutritive functions, and the food out of which
the nutrient products are to be organized.
Newly born children should not be washed in soap and
water, turning them over and over for this purpose, and
then dressed in a fancy suit, including a shirt, two or three
petticoats, a frock and apron, etc. On the contrary, they
should be "wiped gently with a soft rag wrung out of warm
water; and if the child is delicate, a little spirit may be
added, which will stimulate the skin and improve the circu-
lation. It should then be starched behind the ears, around
the neck, armpits, groin, gentals, and nates; after which, if
the weather is cold, it should be wrapped in a sort of man-
tle, or blouse, made of Canton flannel, and placed in bed
beside its mother, not with its head on a pillow, but so ad-
just its wrapper as to let its head rest a shade lower than
its body. The reason for this is obvious : for six months
the new comer has been standing on its head, and a sudden
revolution in this respect may be very hurtful, if not fatal,
to him; and for the first month; whenever he is taken up,
he should be held by the feet, and let the blood gravitate
freely to his head for an instant. If this is done, children
will rarely die of trismus nescentium. As soon as the
mother has sufficiently recovered from her exhaustion to do
so safely, the child should be put to the breast. If there is
milk enough for it at the start, which is often the case, it is
well. One pint of its mother’s milk per day will suffice, un-
til it is eight or ten days old. If the mother has no milk,
then a wet nurse, whose milk is not more than four or five
months old, will answer. But, if the child is left motherless,
or she is in any way incapacitated to nourish it, and a wet
nurse of good health, and otherwise acceptable, cannot be
procured, then artificial milk must be resorted to. We com-
mend the following, originally published in the American
Journal of Niedical Sciences, for July 1858, from the pen
of Wm. Henry Cumming, M. D., formerly of Georgia, now
of Toronto, Canada West. Dr. Cumming’s commanding
ability and educational qualifications fit him, in an eminent
degree, for the investigation of this subject, one of the most
important, in our opinion, connected with the subject of
medicine. The physician has but half done his duty when
he cures disease: the other half, and, perhaps, the greatest
of the two, is to prevent it. The labors of Dr. C. are the
more praiseworthy,because they are addressed to that help-
less period of human life, usually left to chance, ignorance,
and superstition^
Normal Lactation of the Human Race.—In vigorous wro-
men the secretion of milk is copious. And this large amount
is indicated in the unimpregnated state by the great devel-
opment of the mammary glands. In no animal w’ith which
we are acquainted, is there a larger promise in this respect.
The amount ordinarily furnished by a good nurse is from
one and a half to two quarts daily, or from four to five
pounds. But cases often occur in which two children re-
ceive abundant supplies from one mother, involving a secre-
tion of eight pounds at least. An infant three months old
will take from forty-eight to sixty-four fluid ounces daily, in
six or eight half pint doses. During the first year, therefore,
he will take from 1000 to 1300 lbs.
What is the composition of this milk? Without entering
into long and tedious details, it may be simply said that by
the latest and apparently the most exact analysis, its compo-
sition is:—
(Butter 20.76	1AAf. ...	(Butter 20.76 lbs.	1QAft 1k_	(Butter 27 lbs.
Casein 14.34	?S; I Casein 14.34 “	J Casein 18# “
Sugar 75.02	I Sugar 75.02 “	rAntfdn	I Sugar 97^ “
Water 889.88	contain	[water 889.38 “	contaln	[water 1157 “
In 1000 lbs. of milk there are 1.6238 lb. of salts, or 26
ounces, of which 0.5736 lb., or 9 ounces, are phosphate of
lime.
In 1,300 lbs. of milk the salts amount to 2.1 lbs., or 33£
ounces, of which 12 ounces are phosphate of lime.
It thus appears that, during the first year, the child re-
ceives from 110 to 113 pounds of dry solids. He may thus
readily gain 15 or 20 pounds in weight (implying less than
three pounds of dry solids), and yet have a large residue,
from 107 to 140 pounds, to be expended in the production
of heat and in the activity of an energetic vitality. A child
thus nourished, can make teeth and bone without difficulty ;
his functional activity need never be suspended for want of
material; atmospheric changes may be successfully resisted,
and zymotic diseases will have little power.
* * * * * * * * *
The cases in which natural lactation fails are so numerous
as to excite the deepest concern. Human milk can seldom
be obtained, and none of the usually employed substitutes
ordinarily succeed. Is it then too much to hope that physi-
cians will give serious attention and thoughtful considera-
tion to a plan offering a substitute for human milk scientifi-
cally correct and practically successful?
From what has been said of the adaptation of milk by its
peculiar and admirable constitution to the wants of the
young animal, it follows, of necessity, that nothing but milk
can be proposed as a substitute for the natural food. The
only kind that in this country can be readily and certainly
obtained, is that of the cow. But the various kinds of milk
are not identical in composition, being adapted to the differ-
ent degrees of development at birth of the young. The
ruminants are the most advanced in this respect, the human
infant is far behind. It cannot then be supposed that milk
adapted to the stomach of a calf would suit that of a new-
born child. Common observation agrees with chemical an-
alysis in declaring that there is too much casein in the milk
of the cow to be tolerated by the child. This is a well-
known fact, and every one waters the milk. But “ how
much water must be used?” and “will watering do no
harm ?” are questions to be answered only by careful study
of the milk, and close observation of its effects upon the
child.
(Butter........... 38.59	(Butter... 20.76
Cow’s milk,	j Casein........ 40.75	While	human J Casein.......14.34	✓
contains	] Sugar......... 53.87	milk	contains ] Sugar... 75.02
( Water...........866.69	(Water...889.88
Cow’s milk, therefore, contains nearly three times as much
casein as human milk, but less than twice as much butter.
In cow’s milk the butter is to the casein as 100 to 105 ; in
human milk, as 100 to 70. If then, by dilution, we reduce
the butter to 20.70, we shall have 21.92 of casein, or 50 per
cent, more than in human milk. With such an excess of
casein we cannot hope to succed. The stomach of the child
cannot digest it, and it will thus pass through the intestinal
canal, irritating as it goes. Debilitating diarrhoea and, per-
haps, vomiting will occur, and the attempt fail. This is the
usual experience of those who use cow’s milk for infants,
and often leads to the abandonment of milk and the substi-
tution of farinaceous food.*
If, by a further dilution, we reduce the casein to 14.34,
we have only 13.58 of butter, or less than two-thirds of the
proper proportion. Such milk may, for a season, seem to
suit the child, but before long it will be found that he does
not thrive. The reason is plain. The right proportion of
butter is 20.76 ; this warms a child, and supplies nervous en-
ergy. But by withholding one-third you lower the temper-
ature of the body, and deprive the nervous system of one-
third of the special nerve-food, the indispensable lecithin.
What wonder, then, that in a short time pallor and langour
supervene, and the health evidently declines. Continue this
food, and there is one result—starvation. Restore the full
supply of butter, and, if matters have not gone too far for
recovery, warmth and energy will gradually return, the
downward progress be stayed, and vigor replace debility.
It is thus evident that by no mode of dilution can ordi-
nary cow’s milk be made a substitute for human. There
* The food of infants should be all absorbed. During the foetal state,
the food of the body is obtained by endosmosis from the mother’s blood.
During the period of lactation, it comes from the mother’s milk. In this
milk there is no refuse matter: all is absorbed and passes into the circula-
tion. No portion of the milk passes from the stomach, through the intes-
tinal canal, to the rectum. The feces of infants are the excretions of the
intestinal canal and its various glandular appendages. The lower part of
the canal may thus be considered as the excreting duct of the liver, pan-
creas, and the glands of the mucous surface.
What wonder, then, that this duct should be irritated by the presence
of foreign bodies (lumps of curd, starch granules, etc., etc.,) and that this
irritation should continue so long as these foreign bodies remain in con-
tact with its surface ?
No fact is more interesting and important than this, that persistent diar-
rhoea often ceases at once when suitable food is given. By suitable food
•we mean food that is all absorbed. Three or four hours after the first
dose of such food, the diarrhoea ceases entirely.
Bearing this truth in mind, let us never expectjiealth so long as foreign
substances are found in the feces of infants. Even small and smooth par-
ticles of curd produce great irritation, and, in many cases, the ingestion of
such food produces almost immediately a discharge of mucus holding in
suspension these offending substances.
will be, in every case an excess of casein, or a deficiency of
butter. So long as the butter is to the casein as 100 to 105,
instead of as 100 to 70, so long must dilution fail to adapt
it to the wants of the child. But if this original proportion
could be changed to that existing in human milk, we might
have hope of success. And we proceed to show how this
may be done.
If we leave at rest for four or five hours ordinary cow’s
milk, and then remove and examine the upper third, we find
in it 50 per cent, more butter than it at first contained. In
round numbers, its butter is no longer to its casein as 100 to
105, but as 150 to 105, or as 100 to 70. If then, by dilu-
tion of this milk, we reduce the butter to 20.76, we have
14.34 of casein, as in human milk.
Another, and, in some respects, a better mode of obtain-
ing the same result is by using the latter half of the milk
furnished by the cow. The former half contains 22.18 of
butter to 41.63 of casein, while the latter half has 54 of
butter to 38 of casein. Here, again, the right proportion
exists, and, by proper dilution, may be made most accurately
to resemble in its chemical constitution human milk.
To show the accuracy of this imitation, let us prepare
some of this milk by the addition of sugar and water. Its
actual composition is butter 54, casein 38, sugar 53, and
water 855. By adding sugar 142 and water 1458, we have
butter 54, casein 38, sugar 185, and water 2313. Reducing
this to thousandths (that we may compare it with the com-
position of human milk, as previously given), we have but-
ter 30.77, casein 14.61, sugar 75, and water 889.62. The
difference is unworthy of notice.
Not only may ordinary human milk be thus closely imi-
tated, but the colostrum also. To do this, during the first
month of the child’s life, we must use milk containing from
75 to 80 thousandths of butter, or from 94 to 107 per cent,
more than the ordinary milk of the cow. This exceedingly
rich milk may be obtained by taking the upper eighth in-
stead of the upper third of milk left to repose for four or
five hours. It may also be obtained by using the last tenth
of the milk furnished by the cow. In the following sched-
ule the milk of the first month is of this peculiarly oily kind.
It will be seen from this schedule, that, by the gradual
diminution of w’ater, an attempt is made (in imitation of
the natural process) to adapt the food to the growing energy
of the child. It will, of course, be understood by every
practitioner that, in this schedule, age is used to indicate
development. Some children are two or three months behind
their age, and must, be fed accordingly. In general, it is
better to begin witli the milk more diluted than the age and
development would seem to indicate, and then gradually in-
crease its strength. It is better that the food should be in-
sufficient than that it should be indigestible.
SCHEDULE.
For a Child from From Ordinary Take Top Milk. Add Water. Making Food.
Cow's Milk.
2	to 10 days old,	IX quarts,	IX gills,	3X gills,	4X gills.
10 to 20	“	lx	“	IX	“	4X	“	6
20 to 30	“	2	“	2X	“	6	“	8X	“
1	to IX months, IX “	3 “	6X “	9X “
JX to 2	“	IX	“	3X	“	7	“	10X	“
2	to 2X	“	IX	“	4	“	7X	“	12X	“
2Xto 3	“	IX	“	4X	“	7X	“	12	“
3	t0 ?X	“	IX	“	5	“	7X	“	13X	'•
3Xto 4	2	5X “	7X “	13
4	to 4X	“	2X	“	6	“	7X	“	13X	“
4X to 5	“	2X	“	6X	“	7X	“	14	“
5	to 6	“	2X	“	7	“	7	14
6	to 7	“	2X	“	7X	“	6X	“	14	“
7	to 8	“	3	“	8	“	6	“	14	“
8	to 9	“	—	SX	“	6	“	14X	“
9	to 10	“	—	“	8X	“	6	“	14X
10	to 11	“	_	“	8X	“	6	“	14X	“
11	to 12	“	—	“	9	“	5X	“	14X	“
12	to 15	“	—	“	9/	“	5X	“	14X	“
15	to 18	“	—	“	9X	“	5	“	14X	“
18 onward “	—	“	10	“	5	“.	65	“
Eight ordinary teaspoonfuls equal one gill; six equal
three quarters of a gill; four equal half a gill; and two
equal a quarter of a gill.
BRIEF DIRECTIONS FOR FEEDING INFANTS.
The cow should be healthy, from four to ten years old.
Young milk is best for infants; a new milch cow is best.
When the milk is four or five months old, younger milk
should be sought;—it is much better for the child.
The cow should be well fed on grass or hay. No grain,
or meal, or slops, should be given to her. Grass or hay,
water and salt, should be her only food.
She must not be exposed to cold. Well housed in winter.
The milk must stand four or five hours in a cool place.
The top milk should then be taken for the child.
It should then be mixed with cool water; this water
should be soft and pure ; hard water should be avoided.
White sugar should be used for sweetening it; it should
be made a very little sweeter than ordinary cow’s milk.
The child should be fed from a bottle.
The child should be fed ouce in three or four hours.
The milk, when prepared, should be kept in a cool place.
In warm weather it may be prepared twice a day.
Bottles, pans, etc., must be kept sweet and clean.
The milk should be given blood-warm.
The infant should take this food by suction. For this
there are several reasons. 1st. It is the natural mode ; 2d.
We cannot otherwise administer the food at so uniform a
temperature; 3d. We cannot otherwise secure a free flow of
saliva ; 4th. We may thus feed the child in the natural pos-
ture ; the recumbent. There is less danger of regurgitation,
and he sinks to sleep quietly after feeding, if the time for
sleeping has come.
An eight ounce vial, with a quill rolled in a long strip of
Swiss muslin for a stopper, is the best arrangement for clean-
liness and convenience. Tubes having narrow passages can-
not be readily cleansed.
A child ten days old will take about thirty-two ounces
daily, in eight four ounce doses. The doses will increase in
size and somewhat diminish in number, so that in three
months seven eight ounce doses are usually taken. The
milk should be given at regular intervals; the good effects
of method and strict regularity in this matter are very appa-
rent. The child should be early trained to pass six or eight
hours at night without feeding. The temperature should
be from 100 degrees to 104. The child should not be al-
lowed to take the milk too rapidly; ten or fifteen minutes
should be given to each dose. The stomach will not then
be too much distended, the liquid part being quickly ab-
sorbed.	,
This food thus administered may well be styled artificial
human milk. In chemical composition, it most closely re-
sembles the natural secretion of mammary glands of vigor-
ous, healthy women, and it offers to the child all that he
needs for growth, development, warmth, and activity. A
careful observation of its effects for several years has led to
the conviction that it leaves nothing to be desired, and that,
on this food, an infant may be reared with admirable re-
sults. And by this we mean that health, uninterrupted
health, with vigor and energy of the bodily functions, may
be regarded as the natural result of the use of this food.
We mean that gastric and intestinal disorders do not follow
its use, as they so often do that of the milk of mothers. We
mean that under its use dentition will be ordinarily a pain-
less process, and that the teeth will be strong and durable.
Believing that a large proportion of the sickness and death
of infants is the result of insufficient and improper food, we
feel sure that, by the use of this artificial milk, the health
and lives of tens of thousands might be annually preserved.
We believe that if generally used the influence upon the
next generation would be evident in a visible increase of
health and vigor. In our own household it has proved of
priceless value, and we desire that other households may
share the benefit.	Wm. Henry Cumming, M. D.
				

